# Student Behavior Recognition System for the Classroom Environment Based on Skeleton Pose Estimation and Person Detection

**DOI:** 10.3390/s21165314

**Published:** 2021-08-06

**Authors:** Feng-Cheng Lin, Huu-Huy Ngo, Chyi-Ren Dow, Ka-Hou Lam, Hung Linh Le

**Affiliations:** 1 Department of Information Engineering and Computer Science, Feng Chia University, Taichung 40724, Taiwan; crdow@mail.fcu.edu.tw; 2 Faculty of Information Technology, Thai Nguyen University of Information and Communication Technology, Thai Nguyen 250000, Vietnam; nhhuy@ictu.edu.vn; 3 GEO Informatics Inc., Taichung 407, Taiwan; houghost@geo.com.tw; 4 Faculty of Automation Technology, Thai Nguyen University of Information and Communication Technology, Thai Nguyen 250000, Vietnam; lhlinh@ictu.edu.vn

**Keywords:** action recognition, deep learning, object detection, skeleton pose, student behavior

## Abstract

Human action recognition has attracted considerable research attention in the field of computer vision, especially for classroom environments. However, most relevant studies have focused on one specific behavior of students. Therefore, this paper proposes a student behavior recognition system based on skeleton pose estimation and person detection. First, consecutive frames captured with a classroom camera were used as the input images of the proposed system. Then, skeleton data were collected using the OpenPose framework. An error correction scheme was proposed based on the pose estimation and person detection techniques to decrease incorrect connections in the skeleton data. The preprocessed skeleton data were subsequently used to eliminate several joints that had a weak effect on behavior classification. Second, feature extraction was performed to generate feature vectors that represent human postures. The adopted features included normalized joint locations, joint distances, and bone angles. Finally, behavior classification was conducted to recognize student behaviors. A deep neural network was constructed to classify actions, and the proposed system was able to identify the number of students in a classroom. Moreover, a system prototype was implemented to verify the feasibility of the proposed system. The experimental results indicated that the proposed scheme outperformed the skeleton-based scheme in complex situations. The proposed system had a 15.15% higher average precision and 12.15% higher average recall than the skeleton-based scheme did.

## 1. Introduction

Human action recognition is a challenging and attractive research topic in computer vision. It can be applied in various applications, such as video understanding, intelligent surveillance, security, robotics, human–computer interactions, industrial automation, health care, and education [1,2,3,4]. In spite much research work in this domain, many challenges in action recognition have remained unresolved. Viewpoint variation, occlusion, body size variation of subjects, spatiotemporal localization of actions, interclass and intraclass variation, etc., are among those challenges [5]. In the classroom environment, student behaviors can be recorded and analyzed to evaluate teaching quality and student attitudes. Thus, many studies have used human action recognition to recognize student behaviors. However, most of these studies have focused on one specific action of students, such as hand raising gestures [6,7,8,9], sleep gestures [10], and yawning behavior [11,12,13,14]. Therefore, an efficient system that recognizes student behaviors accurately is required.

The skeleton data representation of human poses in videos is a popular technique for action recognition [5,15,16,17,18,19]. In this technique, the main task is to identify the skeleton data, including the detailed location of joints. However, these studies usually use RGB deep images captured by the Microsoft Kinect sensor [20] as the input images of their action recognition systems. Many difficulties arise in the broad implementation of the aforementioned method due to the replacement of the input camera in the classroom. To resolve this issue, the OpenPose framework [21] is used to estimate two-dimensional (2D) human poses [22,23,24]. However, the limitation of this approach is that incorrect connections occur in pose estimation in highly crowded areas such as classrooms.

Therefore, this paper proposes a student behavior recognition system based on pose estimation and person detection. The input images of this system were consecutive frames captured from a classroom camera. Three main processing steps were performed: the collection and preprocessing of skeleton data, the extraction of features, and the classification of behaviors. First, skeleton data were collected and preprocessed. An error correction scheme based on the pose estimation and person detection techniques was proposed to decrease incorrect connections in the skeleton data. In addition, several joints in the skeleton data were removed because they had weak effects on behavior classification. Second, feature extraction was performed to generate feature vectors that represent human postures. The adopted features included normalized joint locations, joint distances, and bone angles. Finally, behavior classification was conducted to recognize student behaviors. A deep neural network was constructed to classify the actions. Furthermore, the proposed system can identify the number of students in a classroom. The main contributions of this paper are as follows:

- A student behavior recognition system was proposed for the classroom environment;
- A new error correction scheme was proposed that combines the pose estimation and person detection techniques. This scheme decreases incorrect connections in the skeleton data;
- The feasibility and efficiency of the proposed scheme and system were verified.

The remainder of this paper is organized as follows. Section 2 presents a literature review of relevant studies and technologies. Section 3 describes the design and architecture of the proposed system. Section 4 details the system implementation and system prototype. Section 5 presents the experimental results. Finally, Section 6 provides the conclusions of this study and directions for future research.

## 2. Related Work

### 2.1. Behavior Detection in the Classroom

The hand raising gesture is the most basic interaction method used by students in classrooms; therefore, numerous studies have focused on detecting this action. Si et al. [8] proposed a region-based fully convolutional network to detect hand raising gestures. A feature pyramid was integrated into their model architecture to improve the detection of low-resolution hand raising gestures. Zhou et al. [9] presented an algorithm for recognizing hand raising gestures. This recognition approach involves three tasks: hand raising detection, pose estimation, and heuristic matching. In addition, it efficiently resolves two major challenges associated with complex scenes: low resolution and motion distortion. Liao et al. [7] proposed a method that involves two stages, namely pose estimation and hand gesture recognition, for identifying hand raising gestures. The aforementioned study analyzed the features of the arms, including the shoulders, elbows, and wrists, which are the main features for identifying hand raising gestures. Jesna et al. [6] introduced a lightweight and efficient method of detecting hand raising gestures. They analyzed the edge structures of the hand by using skin color segmentation and Gaussian filtration techniques.

Researchers have also detected various other student behaviors, such as standing up, sleeping, and yawning. For example, Li et al. [10] presented a method for sleep gesture detection in classrooms. They incorporated a feature pyramid in their model architecture and used a local multiscale testing algorithm for detecting low-resolution gestures. Wang et al. [11] proposed a method for detecting the yawning gestures of students in classrooms. These authors integrated a feature pyramid into their model architecture and used mouth fitting to reduce false detections. Zheng et al. [13] proposed a detector named GestureDet to detect several student behaviors in classrooms, including hand raising, standing up, and sleeping. GestureDet is lightweight and can be efficiently run on embedded devices, such as Nvidia Jetson TX2. Zheng et al. [14] presented an intelligent system for analyzing student behaviors in classrooms. This system can detect three student behaviors: hand raising, standing up, and sleeping. The faster region-based convolutional neural network (R-CNN) model was improved and used in the aforementioned study, and a scale-aware detection head was developed to detect objects of different sizes. Yu et al. [12] developed a behavior measurement system for detecting and analyzing student behaviors in classrooms. This system can detect several student behaviors, such as hand raising, standing up, sitting down, sleeping, and whispering. Microsoft Kinect devices were used to collect the input images for the aforementioned system.

### 2.2. Human Pose Detection

Human pose detection is an attractive research topic in computer vision. The result of human pose detection is the skeleton data, which include detailed joint locations. Two types of skeleton data are available: 3D and 2D skeleton data. Qiang et al. [25] proposed a novel approach for human pose estimation using image sensor data. This method combines convolutional pose machines (CPMs) with GoogLeNet. The first stage of the CPMs directly generates a response map of each human skeleton’s key points from the images. Cao et al. [21] presented a real-time approach (OpenPose) to detect the 2D pose of multiple people in an image. This method uses a nonparametric representation, called Part Affinity Fields (PAFs), to learn to associate body parts with individuals in the image. Jin et al. [26] proposed a novel differentiable hierarchical graph grouping method to estimate multiperson poses. This method could learn the graph grouping in a bottom-up multiperson pose estimation task. They investigated a new perspective of the human part grouping problem as a graph clustering task. Dai et al. [27] presented a novel relation-based skeleton graph network (RSGNet) for multiperson pose estimation in crowded scenes. They introduced a skeleton graph machine to enforce the constraint of the human body structure during the joints’ inference for accurate pose estimation.

To collect 3D skeleton data, RGB deep images are captured by the Microsoft Kinect sensor. This method is one of the most popular to estimate 3D human pose [5,16,18]. The method converts 2D image detections from multiple camera views into 3D images [28,29,30]. Li et al. [30] proposed a 3D human pose detection approach using multiview cameras. This method uses the OpenPose framework to obtain 2D joints in every image and combines Mask R-CNN [31] to segment the human semantics. Then, an assembling method was proposed to select the correct 3D pose from the multiview 2D joints by joint semantic meanings. Slembrouck et al. [32] presented a novel multiview video-based markerless system for 3D human pose estimation. This method converts 2D skeleton detections from multiple camera views into 3D skeletons. The OpenPose framework was used to obtain 2D joints.

### 2.3. Action Recognition Using Skeleton Data

Various approaches have been proposed for human action recognition using skeleton data [5,16,17,33]. Most of these approaches involve the use of three-dimensional (3D) skeleton data. Agahian et al. [5] introduced a framework based on 3D skeleton data for human action recognition. The main techniques in this framework include pose estimation and encoding. Cippitelli et al. [16] presented a system based on 3D skeleton data for recognizing human activity. This system can be used for monitoring elderly people in the home environment. In the aforementioned system, a feature vector is generated through the extraction of key poses, and a multiclass support vector machine is used to classify human activities. Khaire et al. [3] proposed a human activity recognition approach based on convolutional neural networks (CNNs) and multiple vision cues. They also presented a method for creating skeleton images based on three features: a motion history image, depth motion maps, and skeleton data. Luvizon et al. [33] presented a framework based on skeleton information for recognizing human actions. Sets of spatial and temporal local features were extracted from subgroups of joints. Subsequently, the vector of locally aggregated descriptors (VLAD) algorithm and a pool of clusters were used to aggregate the local features into several feature vectors. Negin et al. [19] proposed a bag-of-poses framework based on 3D skeleton data for recognizing human actions. In this framework, each action is represented by a set of spatiotemporal poses. The pose descriptor comprises two sections: the normalized coordinate of the skeleton joints and the temporal displacement of the joints.

RGB deep images are used to create 3D skeleton data. Therefore, systems based on such data require a special camera, such as that on the Microsoft Kinect. Consequently, several studies have used 2D skeleton data for human action recognition. Aubry et al. [22] presented a human action recognition method based on 2D skeleton data. This method involves using the OpenPose framework to estimate human poses. Noori et al. [23] introduced a new approach for recognizing human activity. This approach involves using the OpenPose framework to extract key points in an image. To classify human activities, the aforementioned authors developed an LSTM-RNN model by combining the long short-term memory (LSTM) model with the recurrent neural network (RNN) model. Schneider et al. [24] presented a method for recognizing human gestures. In this method, the OpenPose framework is used to detect human poses, and the one-nearest-neighbor classifier is used to classify the detected poses.

## 3. Model of the Behavior Recognition System Used in This Study

### 3.1. System Architecture

**Figure 1:**
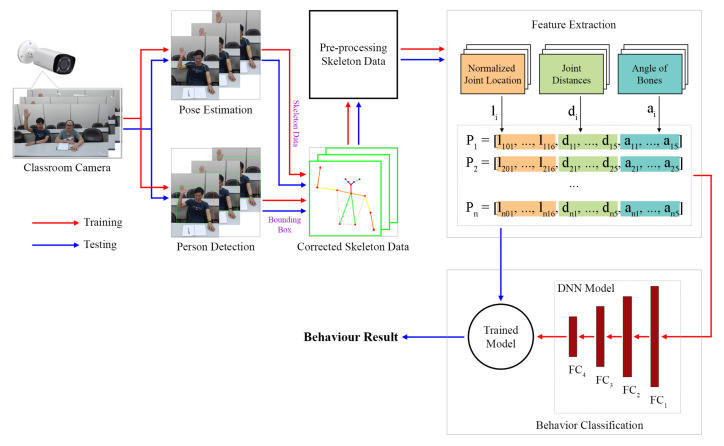
System overview.

Figure 1 displays an overview of the behavior recognition system used in this study. In this figure, the red and blue arrows denote the training and testing processes of the system, respectively. The input images of this system were successive video frames captured by a classroom camera. In the aforementioned system, three main processing steps are performed: the collection and preprocessing of skeleton data, the extraction of features, and the classification of behaviors.

Two main processes were involved in collecting the skeleton data: pose estimation and person detection. The collected skeleton data were corrected using a scheme proposed in this paper, which is an error correction scheme that combines the pose estimation and person detection schemes. The proposed scheme reduces the incorrect connections in the skeleton data. The preprocessed skeleton data were used to remove several joints that had weak effects on behavior classification. Next, feature extraction was performed to generate feature vectors that represent human postures. The features used included normalized joint locations, joint distances, and bone angles. These three feature vectors were concatenated to create a final feature vector, which was used for the training and testing processes related to the proposed system. Finally, behavior classification was conducted to recognize student behaviors. A deep neural network (DNN) was developed for training the classification model. This trained model was used for testing the proposed system. This system can also identify the number of students in a classroom.

### 3.2. Human Pose Estimation

Human pose estimation is a highly critical step because it directly affects the quality of a behavior recognition system. Detailed skeleton data, including detailed joint locations, were used in this study. Two types of skeleton data are available: 3D and 2D skeleton data. To collect 3D skeleton data, RGB deep images are captured by the Microsoft Kinect [20] sensor, which are the input of behavior recognition systems [5,16,18,33,34]. The OpenPose framework [21] is one of the most popular approaches for detecting 2D human poses [22,23,24].

**Figure 2:**
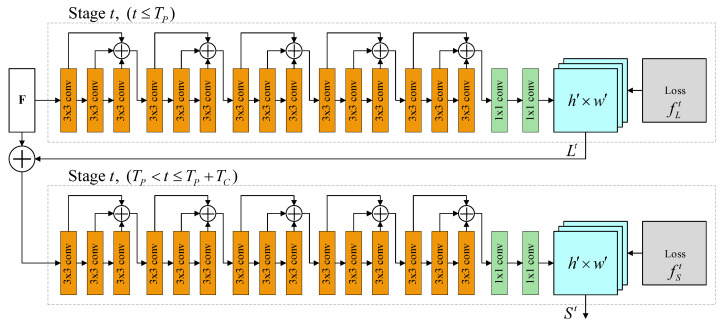
Network architecture of OpenPose [21].

**Figure 3:**
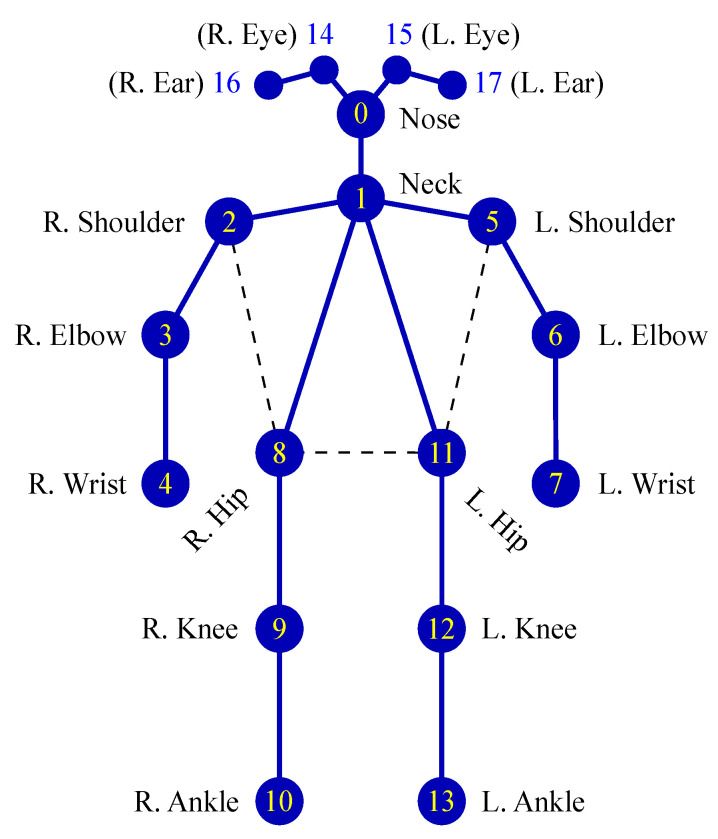
Key points for human poses according to the COCO output format (R/L: right/left).

The proposed system uses consecutive frames captured by a classroom camera as the input images. Moreover, the results of human pose estimation are 2D skeleton data. Therefore, the OpenPose framework was used to develop the proposed system. Figure 2 illustrates the network architecture of OpenPose. OpenPose is a multistage CNN that contains two main flows. The first flow predicts part affinity fields (PAFs) that encode part-to-part association ($L^{t}$), and the second flow predicts confidence maps ($S^{t}$). A greedy inference algorithm was used to parse the PAFs and confidence maps to produce 2D key points (joints) for all people in the image. OpenPose generates skeletons, including the locations of 18 joints for each human pose, according to the COCO output format, as displayed in Figure 3 [35]. The skeleton data cover the joints of the head, neck, arms, and legs. Each joint location is described in the image by *x*-coordinate and *y*-coordinate values; thus, each piece of skeleton data comprises 36 features. The skeleton data were used to compute, analyze, and create the input data for the proposed behavior classification model.

### 3.3. Person Detection and Skeleton Data Preprocessing

#### 3.3.1. Person Detection

Object detection has become an increasingly important technique in the field of computer vision, and it can be broadly used in various applications. Due to recent advances in deep learning and CNN architectures, CNNs are widely used in object detection, especially person detection. This phenomenon has led to the development of many high-accuracy object detection methods, such as Faster R-CNN [36], SSD [37], YOLOv3 [38], YOLOv4 [39], and Mask R-CNN [31]. The Mask R-CNN scheme proposed by He et al. [31] aims to generate bounding boxes and object instance segmentation in the image. This method extends Faster R-CNN by adding a branch for predicting an object mask in parallel with the existing branch for bounding box recognition. Ma et al. [40] proposed a conceptually novel matting-enhanced Mask R-CNN (MMask R-CNN) that extends Mask R-CNN by adding a new mask head to model the pixelwise classification uncertainty with the predicted alpha matte. The trade-off between accuracy and processing time was considered in the selection of the model for designing and constructing the proposed system. On the basis of this consideration, the YOLOv4 model was used to develop the person detection function.

The YOLOv4 model was trained on the COCO dataset [41], and this model could detect objects belonging to 80 classes, including airplane, bicycle, bus, car, and person classes. However, only people were considered in this study; therefore, we used a filter in the output layer to obtain objects belonging to only the person class [42].

#### 3.3.2. The Error Correction Scheme

After collecting skeleton data, the skeletons and bounding boxes of the detected people were determined. However, these skeletons usually contain some incorrect connections, especially in crowds of people. Therefore, an error correction scheme based on the combination of the pose estimation and person detection schemes was proposed in this paper. This scheme eliminates the incorrect connection in skeleton data and is performed in two phases.

In the first phase, a skeleton corresponding to each bounding box of the detected person is identified; that is, the bounding boxes of skeletons are set. In addition, we can count the number of people in a classroom. For these purposes, we propose Algorithm 1 and detailed descriptions in the following:

1. Lines 1 and 2 of Algorithm 1 contain the input and output information of the algorithm. The input comprises two variables: skeletons and boxes_object. The skeletons variable is the skeleton data of people, and the boxes_object variable contains the bounding boxes of the people detected by the person detection scheme. The output of the algorithm comprises two results: boxes_skeleton and boxes_all. The boxes_skeleton variable represents the bounding boxes of the skeletons, and the boxes_all variable represents all the bounding boxes of the people in an image;
2. Lines 3–8 are related to the initialization of the boxes_skeleton variable by using skeleton data;
3. The parameters are set using Lines 9 and 10. The status_skeletons variable is used to consider whether the bounding box of the skeleton has been identified;
4. Lines 11–19 are used to determine the bounding box corresponding to the skeleton. The count_points function counts the skeleton points that belong to a box area;
5. Lines 20 and 21 indicate that a bounding box does not correspond to any skeleton, and this box is added to the boxes_all variable. In other words, this person is detected by the person detection scheme; however, the skeleton of the person is not identified by the pose estimation scheme;
6. Lines 22–26 are used to set the bounding box of a skeleton, and the status of this skeleton is set to false. Finally, the program returns the bounding boxes of the skeletons and all bounding boxes of the people in an image.

**Algorithm 1.** Identifying the bounding boxes of each skeleton.
1.  **Input:** 
$skeletons=\{body_{i}\left\{j:\left(x_{joint\_j},y_{joint\_j}\right),j=\left[0,17\right]\right\},i=\left[0,n-1\right]\}$
     $boxes\_object=\{box_{i}\left(x_{1},y_{1},x_{2},y_{2}\right),i=\left[0,m-1\right]\}$     *n*: number of skeletons
     *m*: number of bounding boxes
2.  **Output:** $boxes\_skeleton=\{box_{i}\left(x_{1},y_{1},x_{2},y_{2}\right),i=\left[0,n-1\right]\}$
     $boxes\_all=\{box_{j}\left(x_{1},y_{1},x_{2},y_{2}\right),j=\left[0,k-1\right]\}$     *k*: number of persons3.  **for** body **in** skeletons:4.   $x_{1}$ = min$\left(x_{body}\right)$5.   $y_{1}$ = min$\left(y_{body}\right)$6.   $x_{2}$ = max$\left(x_{body}\right)$7.   $y_{2}$ = max$\left(y_{body}\right)$8.   boxes_skeleton.append$\left(x_{1},y_{1},x_{2},y_{2}\right)$9.   boxes_all=boxes_skeleton10.  status_skeletons = [True **for** (*i*, item) **in** enumerate(skeletons)]11.  **for** (index_box, box) **in** enumerate(boxes_object):12:         index_max = -113.         number_joints = 014.         **for** (index_body, body) **in** enumerate(skeletons):15.     **if** not(status_skeletons[index_body]):16.        continue17.      **if** number_joints < count_points(box, body):18.        number_joints = count_points(box, body)19.        index_max=index_body20.   **if** index_max == -1:21.         boxes_all.append(box)22.   **else**:23.         status_skeletons[index_max] = False24.         boxes_skeleton[index_max]=box25.         boxes_all[index_max]=box26.   **return** boxes_skeleton,boxes_all

In the second phase, Algorithm 2 is executed to eliminate the incorrect connections in the skeleton data:

1. Lines 1 and 2 of Algorithm 2 contain the input and output information of the algorithm. The input comprises the skeletons and the bounding boxes of the skeletons (boxes_skeleton). The new_skeletons variable is the result returned by the algorithm, and it contains the new skeletons;
2. Line 3 is related to the initialization of the new_skeletons variable;
3. In Lines 4 and 5, the skeletons and their joints are considered;
4. Lines 6–9 indicate that if a joint does not belong to the bounding box of a skeleton, the joint is excluded from the skeleton and all connections of this joint are eliminated. The belong_to_area function is used to determine whether a point belongs to a box area. Finally, the program returns the new skeletons.

**Algorithm 2.** Editing joint connections.
1. **Input:** $skeletons=\{body_{i}\left\{j:\left(x_{joint\_j},y_{joint\_j}\right),j=\left[0,17\right]\right\},i=\left[0,n-1\right]\}$
               $boxes\_object=\{box_{i}\left(x_{1},y_{1},x_{2},y_{2}\right),i=\left[0,m-1\right]\}$
               *n*: number of skeletons
2.   **Output:** $new\_skeletons=\{body_{i}\left\{j:\left(x_{joint\_j},y_{joint\_j}\right),j=\left[0,17\right]\right\},i=\left[0,n-1\right]\}$3.   new_skeletons=skeletons4.   **for** (index_body, body) **in** enumerate(skeletons):5.         **for** joint **in** body:6.     **if** not(belong_to_area(boxes_skeleton[index_body],joint)):7.        new_skeletons.remove(joint)8.        new_skeletons.remove(all connections of joint)9.   **return** new_skeletons

**Figure 4:**
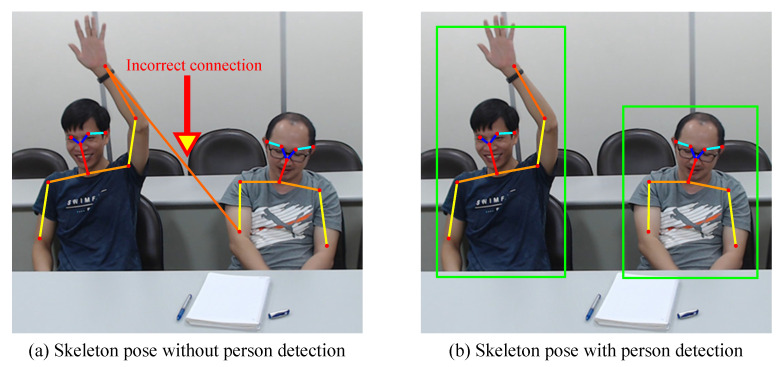
Illustration of the combination of pose estimation and person detection.

Figure 4 illustrates the combination of pose estimation and person detection. Figure 4a illustrates the use of the pose estimation scheme for collecting skeleton data. An incorrect connection exists in the skeleton data. Figure 4b represents how the pose estimation scheme is combined with the person detection scheme to collect skeleton data. The error correction scheme is used to eliminate the incorrect connection.

#### 3.3.3. Preprocessing of Skeleton Data

This study focused on recognizing four major student behaviors: asking, looking, bowing, and boring. These behaviors are described as follows:

- Asking: The student is raising his/her hand. This behavior by a student indicates that the student has a highly positive attitude;
- Looking: The student is looking at the teacher. When looking at the teacher, a student’s back is straight and inclined toward the desk. The student’s head is in a neutral position and in line with the torso. The behavior of looking also indicates the positive attitude of a student;
- Bowing: A student exhibits bowing behavior when writing or reading documents. During such behavior, their head is bowed and they are focused on their documents. This behavior indicates the neutral attitude of a student;
- Boring: When a student is bored, they do not focus on the task at hand. They cross their arms on the table and rest their head on their hands. This behavior demonstrates the negative attitude of a student.

**Figure 5:**
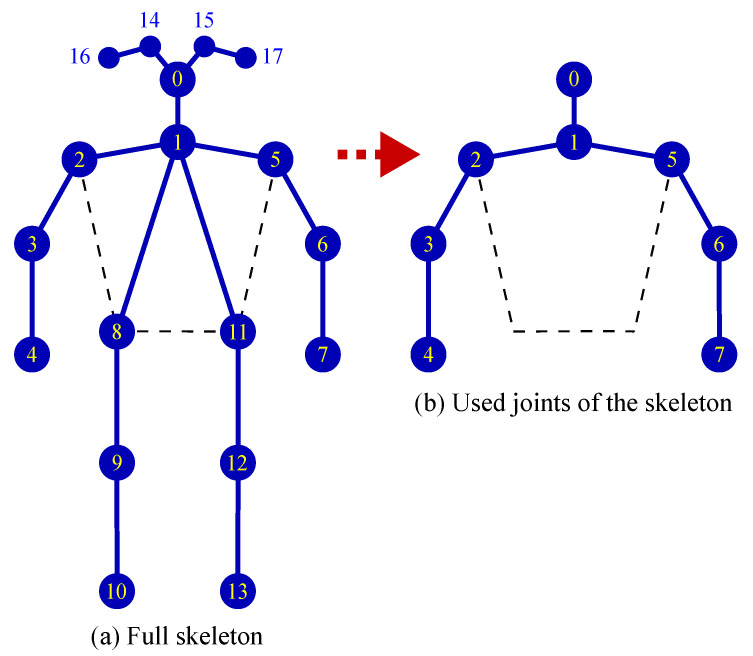
Preprocessing and selection of skeleton joints.

Through behavior analysis, we identified several joints that had weak effects on behavior classification, such as the joints of the eyes, ears, hips, knees, and ankles (Figure 5). Therefore, these joints were eliminated before feature extraction. By eliminating the aforementioned joints, the feature extraction time can be shortened and the incorrect connections in human pose estimation can be reduced.

A classroom environment usually contains groups of people. Therefore, the collected skeleton data may sometimes be incomplete due to the occlusion of different human parts. This phenomenon directly affects the quality of the proposed system. To resolve this issue, a simple moving average technique is used to smooth the skeleton data for reducing the influence of corrupted data [43].

### 3.4. Skeleton Feature Extraction

Skeleton feature extraction is a crucial task in the proposed system. In this task, a vector that represents a human pose is generated. The current study was inspired by several studies on human action recognition [1,16,25]. In the current study, the aforementioned vector was generated by concatenating three component vectors: the vectors of the normalized joint locations, joint distances, and bone angles.

#### 3.4.1. Normalized Joint Location

The first component vector is the joint locations. People occupy different areas in the same frame due to their different distances from the camera; thus, the scales of the joint locations are different. Consequently, the joint locations are normalized using Equation (1), where ($x_{i},y_{i}$) and ($x_{i}^{\prime },y_{i}^{\prime }$) are the original and normalized locations of the $i^{th}$ joint, respectively. Each joint location is described in the image by the *x*-coordinate and *y*-coordinate values; thus, the vector of the normalized joint locations comprises 16 features corresponding to eight joints.

(1) $$\left(x_{i}^{\prime },y_{i}^{\prime }\right)=\left(\frac{x_{i}}{width\_image},\frac{y_{i}}{height\_image}\right);Where:i=\left[0,7\right]$$

#### 3.4.2. Joint Distances

**Figure 6:**
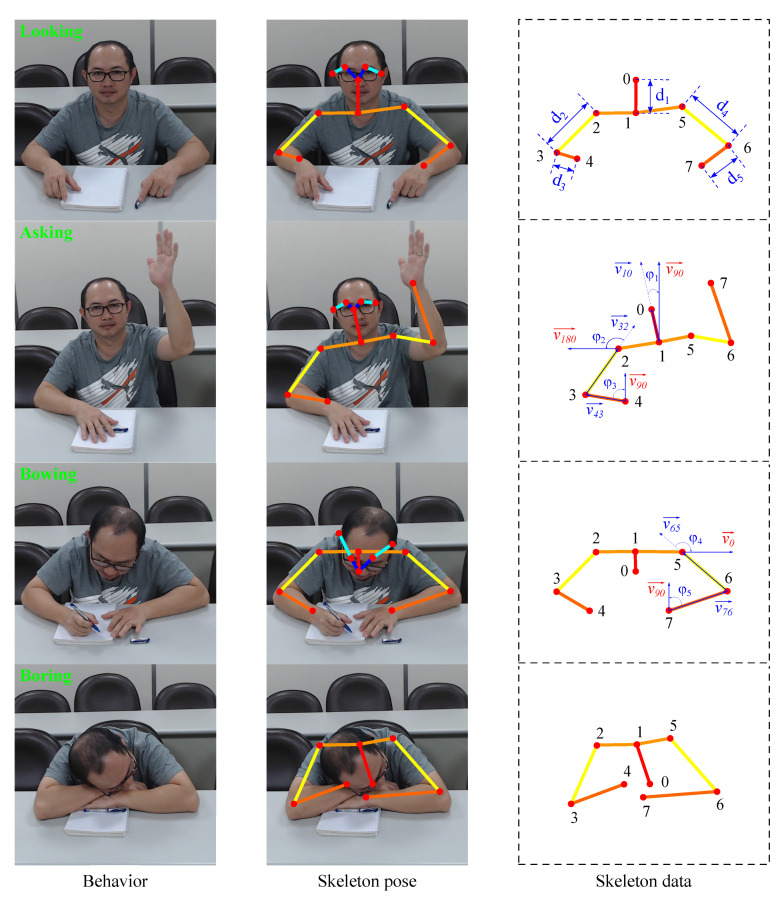
Analysis of the skeleton data.

After the joint locations are normalized, the second component vector is created by calculating the joint distances. The Euclidean distance between Joints A and B is calculated using Equation (2). The vector of the joint distances contains five features corresponding to five distances (from $d_{1}$ to $d_{5}$), as shown in Figure 6. The distance between the shoulder joint and the neck joint is relatively unchanged in every behavior; therefore, this distance is not considered in feature extraction.

(2) $$dist\left(A,B\right)=\sqrt{{\left(x_{B}-x_{A}\right)}^{2}+{\left(y_{B}-y_{A}\right)}^{2}}$$

#### 3.4.3. Bone Angles

The third component vector is for bone angles. This vector has five features corresponding to five angles (from $\varphi_{1}$ to $\varphi_{5}$), as depicted in Figure 6:

- $\varphi_{1}$: the angle between the vector $\vec{v_{10}}$ connecting the neck joint to the nose joint and the vector $\vec{v_{90}}$ at 90° to the horizontal plane;
- $\varphi_{2}$: the angle between the vector $\vec{v_{32}}$ connecting the right elbow joint to the right shoulder joint and the vector $\vec{v_{180}}$ at 180° to the horizontal plane;
- $\varphi_{3}$: the angle between the vector $\vec{v_{43}}$ connecting the right wrist joint to the right elbow joint and the vector $\vec{v_{90}}$ at 90° to the horizontal plane;
- $\varphi_{4}$: the angle between the vector $\vec{v_{65}}$ connecting the left elbow joint to the left shoulder joint and the vector $\vec{v_{0}}$ along the horizontal plane;
- $\varphi_{5}$: the angle between the vector $\vec{v_{76}}$ connecting the left wrist joint to the left elbow joint and the vector $\vec{v_{90}}$ at 90° to the horizontal plane.

### 3.5. Behavior Classification

**Figure 7:**
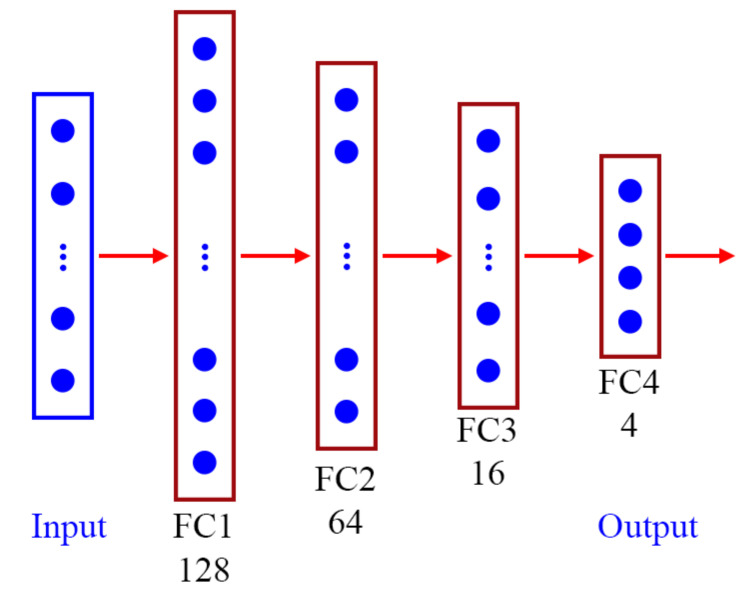
Architecture of the DNN model.

A deep neural network model was constructed to classify student behaviors (Figure 7). This model is a small and simple network that contains an input layer and four fully connected (FC) layers. The input layer is a vector that contains 26 features and represents human posture. Each of the first three FC layers is followed by a rectified linear unit activation function layer and a batch normalization layer. The first, second, and third FC layers contain 128, 64, and 16 neurons, respectively. The final FC layer is followed by a softmax activation function layer, and the final FC layer contains four neurons.

## 4. System Implementation and Prototype

A prototype was designed to verify the efficiency of the proposed system. The prototype was implemented on a 3.70-GHz Intel Core i7 CPU with a 64-bit Windows 10 operating system, 32 GB of RAM, and an NVIDIA TITAN V GPU. The program was developed using Anaconda3 Version 5.2.0 as the integrated development environment, and the programming language was Python Version 3.6.5.

**Figure 8:**
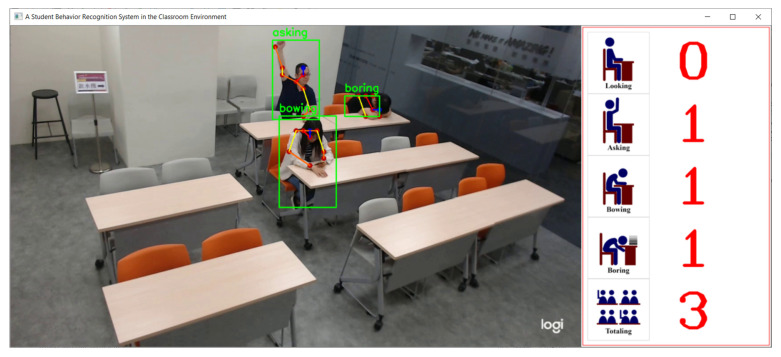
User interface of the proposed system.

Figure 8 displays the user interface of the proposed system. This interface comprises two parts; these parts show the camera view and behavior statistics. The camera view is on the left of the interface and allows users to observe the entire classroom and student behaviors. The behavior statistics part displays the number of occurrences of asking, looking, bowing, and boring behaviors, as well as the total number of people in the classroom.

## 5. Experimental Results

### 5.1. Dataset Description

*Training dataset*: The training dataset contained 11,500 images belonging to four classes: the asking, boring, bowing, and looking classes. The training dataset consisted of two parts: the training and validation datasets, which accounted for 70% and 30%, respectively. The training dataset consisted of selected videos with different parameters, such as imaging angles, light intensity, backgrounds, and the sizes of people in an image. To label behaviors quickly, the selected videos contained only one person in each frame, which corresponded to an action. Table 1 presents the number of images related to each behavior in the training process.

**Figure 9:**
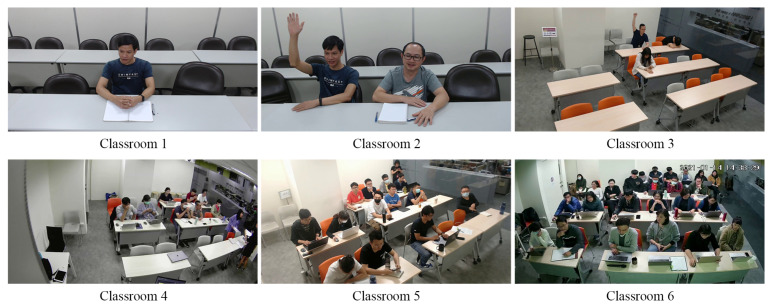
Snapshots of six classrooms.

*Testing dataset*: The behavior recognition scheme is usually affected by factors such as imaging angle, light intensity, crowded scenes, and people occupying different areas in an image. The testing datasets were created for testing different situations. As presented in Table 2, six classroom scenarios were designed to evaluate the proposed scheme under various situations, such as crowded scenes, different camera angles, and people occupying different areas of an image due to their different distances from the camera. The snapshots of the six classrooms are displayed in Figure 9.

### 5.2. Model Training Evaluation

After the large dataset was collected and features were extracted, the deep neural network model was trained. This task is essential and directly affects the quality of the proposed system. This section presents the results of model training.

**Figure 10:**
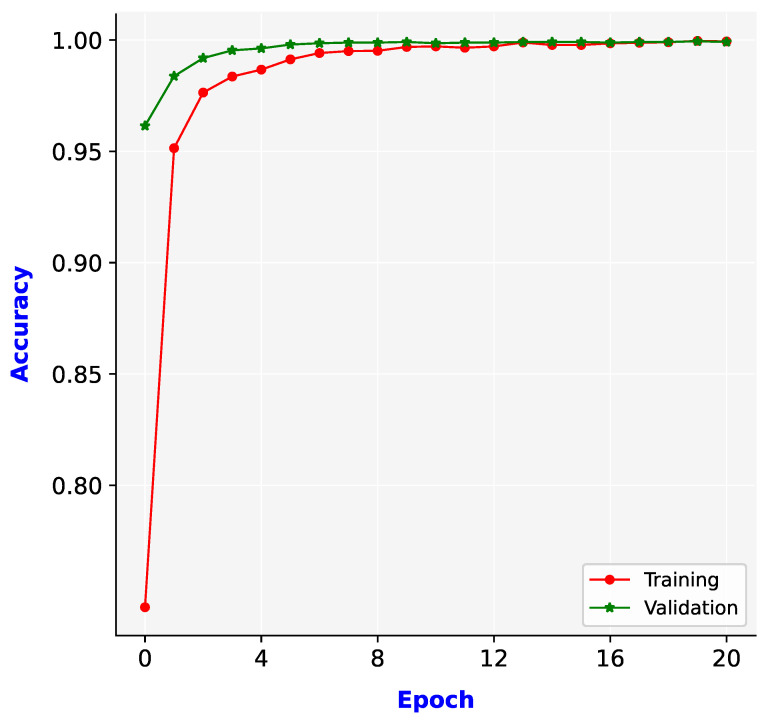
Training and validation accuracies for the constructed model.

Figure 10 illustrates the training and validation accuracy levels achieved for the training dataset. The training and validation accuracy levels improved with each epoch. After 12 epochs, these accuracy levels remained consistently high and reached a saturation level. At the end of 20 epochs, the training and validation accuracy levels were 0.9994 and 0.9991, respectively. These accuracy levels are outstanding and help the proposed system to achieve excellent performance. The trained model was used to evaluate and implement the proposed system.

### 5.3. Comparison of Schemes in Terms of Precision and Recall

The performance of schemes can be evaluated using different metrics, such as precision and recall. Precision and recall are calculated using Equations (3) and (4), respectively.

(3) $$Precision=\frac{True\;Positive}{True\;Positive+False\;Positive}\times 100\%$$

(4) $$Recall=\frac{True\;Positive}{True\;Positive+False\;Negative}\times 100\%$$

We compared two approaches: the skeleton-based scheme and the proposed scheme. The skeleton-based scheme was inspired by three studies on human action recognition [1,16,25]. In the current study, improvements were made to the three processing steps in the aforementioned scheme: the collection and preprocessing of skeleton data, the extraction of features, and the classification of behaviors. The person detection technique was not used in the skeleton-based scheme. In the proposed scheme, the skeleton-based scheme was combined with a person detection technique.

**Figure 11:**
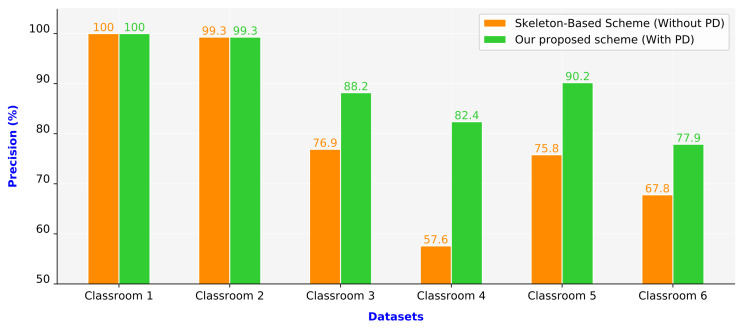
Precision values of the schemes (PD: person detection).

Figure 11 presents the precision values of the two compared schemes. The proposed scheme outperformed the skeleton-based scheme. In the first two scenarios (Classrooms 1 and 2), both schemes achieved consistently high precision because these scenarios represent simple situations. Classrooms 1 and 2 comprised only one or two people per image frame, and the camera was located in front of the people in these classrooms. The remaining four classroom scenarios (Classrooms 3–6) represent complex situations. Classrooms 3–6 were crowded, and the camera was placed in the corner in these classrooms. People occupied different areas in the same image frame due to their different distances from the camera. In Classroom 4, the skeleton-based scheme had the lowest precision value of only 57.6%. Due to most students in this classroom being at the end of the classroom that is far from the camera, people’s sizes looked small. Besides, the camera was placed in the corner in this classroom. Therefore, the human pose estimation had numerous incorrect connections. By contrast, the proposed scheme for Classroom 5 achieved the highest precision value of 90.2% among Classrooms 3–6. We observed that the camera view in this classroom had zoomed in, and students were near the camera. Furthermore, students sat separately and did not overlap each other. In Classrooms 3–6, the proposed scheme exhibited superior performance to the skeleton-based scheme. The proposed scheme had a 15.15% higher average precision than the skeleton-based scheme did.

**Figure 12:**
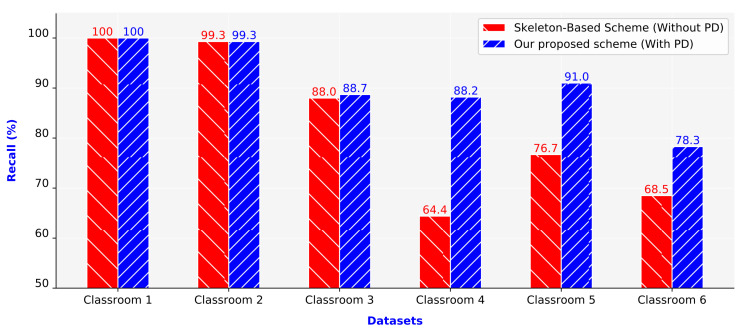
Recall values of the schemes.

Figure 12 presents the recall values of the two compared schemes. Similar to the precision values, the recall values of the proposed scheme were also higher than those of the skeleton-based scheme. The recall values of the two schemes were almost equal in Classrooms 1 and 2 (100% and 99.3%, respectively). For the remaining four classrooms (Classrooms 3–6), the two schemes had considerably different recall values. The recall of the proposed scheme was higher than that of the skeleton-based scheme. The average recall of the proposed scheme was 12.15% higher than that of the skeleton-based scheme.

**Figure 13:**
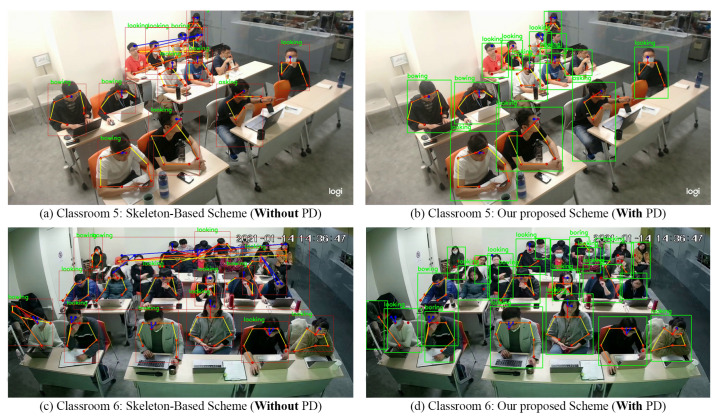
Comparison of the two schemes in Classrooms 5 and 6.

Figure 13 illustrates the pose estimation results of the two schemes for Classrooms 5 and 6. The sample frame of Classroom 5 comprised eight incorrect connections when the skeleton-based scheme was used and no incorrect connections when the proposed scheme was used (Figure 13a,b, respectively). The sample frame of Classroom 6 comprised eighteen and three incorrect connections when the skeleton-based and proposed schemes were used, respectively (Figure 13c,d, respectively). The aforementioned results indicate the outstanding performance of the proposed scheme in complex situations.

### 5.4. Examination of People Detection

We evaluated the people detection accuracies of the two compared schemes. The people detection accuracy is defined in Equation (5), where $N_{correct}$ indicates the number of correctly predicted people and $S_{real}$ represents the total number of real people.

(5) $$Accuracy=\frac{N_{correct}}{S_{real}}\times 100\%$$

**Figure 14:**
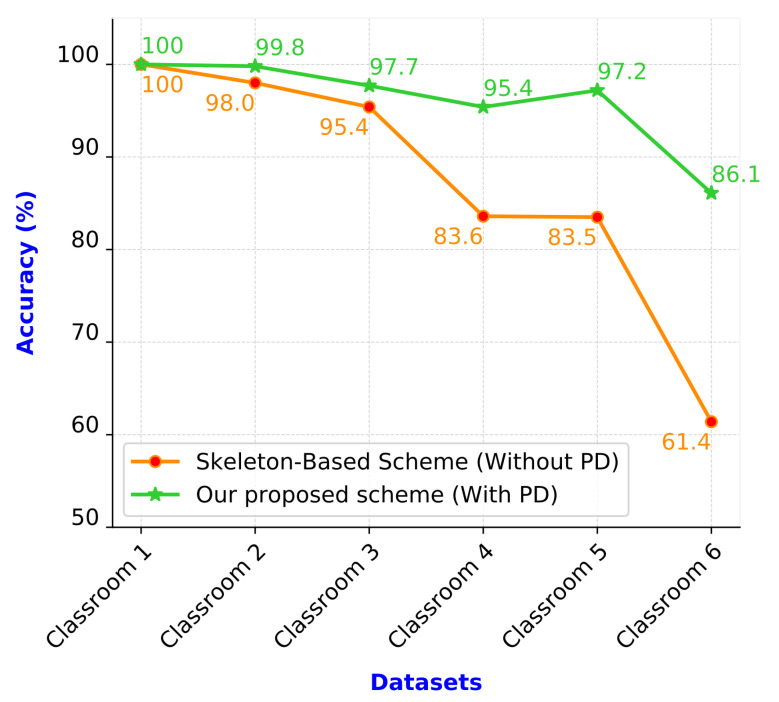
Results of people detection.

Figure 14 presents the people detection accuracies of the two compared schemes. The proposed scheme achieved higher people prediction accuracies than the skeleton-based scheme, especially for Classrooms 4–6. The people detection accuracies of the skeleton-based and proposed schemes were 61.4% and 86.1% for Classroom 6, respectively. Classrooms 4–6 contained large numbers of people per image and crowded people; therefore, numerous incorrect connections were evident in the skeleton data. In the skeleton-based scheme, these incorrect skeleton data were directly used to extract features, detect people, and recognize student behaviors. Consequently, numerous false people detections and false behavior recognitions occurred in the aforementioned scheme. In the proposed scheme, this problem was perfectly resolved using an error correction scheme. The proposed scheme decreases the incorrect connections in the skeleton data. Therefore, the performance of the proposed scheme was significantly superior to that of the skeleton-based scheme.

### 5.5. Comparison of the Average Processing Time

In addition to accuracy, processing time has a considerable influence on system performance. When a system is being constructed and assessed, both accuracy and processing time must be considered because they usually have a trade-off relationship. Table 3 presents the average processing time of the two compared schemes. As presented in Table 3, the skeleton-based scheme had a shorter processing time than did the proposed scheme. The skeleton-based and proposed schemes had processing times of 100 and 335 milliseconds per frame, respectively. In the proposed scheme, the skeleton-based scheme is combined with the person detection technique (YOLOv4). All the processing times obtained in this study were acceptable because the statistics of student behaviors were usually calculated within a few seconds.

### 5.6. Analysis of Monitoring the Classroom

**Figure 15:**
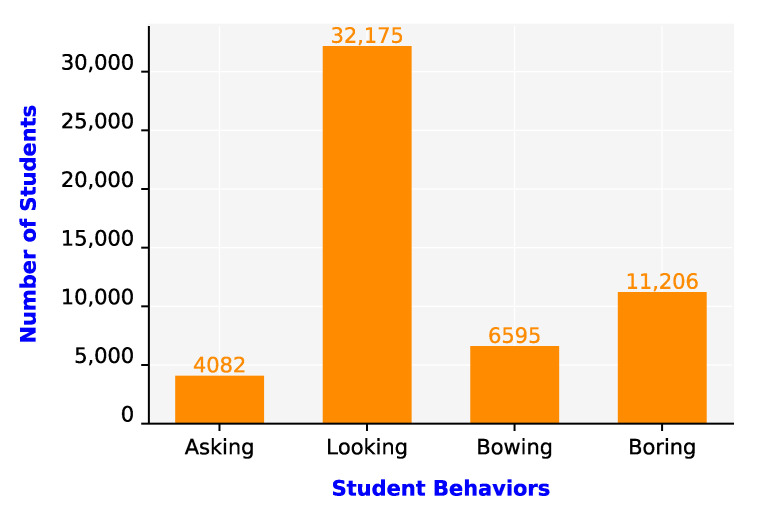
Results of student behavior recognition.

To evaluate the teaching quality and student attitudes, we analyzed the student behaviors in Classroom 6 over 50 min. Image frames were captured at a rate of one frame per second. Figure 15 displays the results of student behavior recognition. As displayed in Figure 15, the largest number of behaviors belonged to the looking category (32,175). This result indicates that a majority of the students had a positive attitude in class. The number of boring behaviors was 11,206. However, some bowing behaviors were incorrectly recognized as boring behaviors. Finally, the numbers of bowing and asking behaviors were 6595 and 4082, respectively.

There is still room for optimization in the future. An error correction scheme based on the combination of the pose estimation and person detection schemes was proposed in this paper. This scheme eliminates the incorrect connection in skeleton data and is performed in two phases. Our proposed scheme has excellent performance. However, a classroom environment usually contains groups of people. Therefore, the collected skeleton data may sometimes be incomplete due to the occlusion of different human parts. This phenomenon directly affects the quality of the proposed system. We will resolve this issue more thoroughly to improve the proposed system in the future.

## 6. Conclusions

We developed and implemented a student behavior recognition system based on skeleton pose estimation and person detection in the classroom environment. This paper also proposed a novel error correction scheme that combines pose estimation and person detection techniques. The proposed scheme reduces the incorrect connections in skeleton data. This study compared the behavior detection performance of the proposed and skeleton-based schemes. The results indicated that the proposed scheme outperformed the skeleton-based scheme in complex situations. The proposed scheme had a 15.15% higher average precision and 12.15% higher average recall than the skeleton-based scheme did. The proposed scheme also achieved higher people detection accuracy than did the skeleton-based scheme. Furthermore, the average processing times of both schemes were acceptable.

There are three points for further research to expand our work that are worth mentioning. Firstly, we can improve the accuracy of hand raising by using multiple cameras in the back of the classroom and adjusting the shooting angle. Secondly, bowing and looking are not easily detectable under different angles and distances, and we recommend that further study can be focused on the angle of the eyes and face after a specific deep photo enhancer. Lastly, we will improve the efficiency of our method and consider more actions, such as chatting, standing, and other actions, by increasing the resolution of the images, for example using a 4 K photograph can help the accuracy of recognition.

This research can give feedback information to the teacher in the class in a period of time. If the teacher provides some interesting content, such as jokes or announces important messages, we can observe whether the students are paying attention to the lecture. Alternatively, a teacher may talk about a subject that is hard to understand; therefore, the students will even become bored or dejected. These are very important messages for teachers in the teaching field.

In the future, the proposed system will be improved through the processing of skeleton data that are incomplete due to the occlusion of different human parts. We also intend to extend the proposed system to the recognition of other actions in various environments. Moreover, we plan to implement the proposed system on an embedded system, such as Jetson AGX Xavier, Jetson Xavier NX, or Jetson TX2.

## Figures and Tables

**Table 1:** Description of the training dataset.

Behaviors	Number of Images for Training	Number of Images for Validation	Total Number of Images
Asking	2450	1050	3500
Boring	1400	600	2000
Bowing	2100	900	3000
Looking	2100	900	3000

**Table 2:** Description of the testing dataset.

Datasets	Number of Images Used	Number of People	Average Number of People per Image	Camera Location
Classroom 1	300	300	1	In front of people
Classroom 2	300	600	2	In front of people
Classroom 3	300	852	2.84	In the classroom corner
Classroom 4	300	2254	7.51	In the classroom corner
Classroom 5	300	4204	14.01	In the classroom corner
Classroom 6	300	7600	25.33	In front of people

**Table 3:** Results of the average processing time.

Methods	Skeleton-Based Scheme	Our Proposed Scheme
Processing time (milliseconds per frame)	100	335

## Data Availability

Not applicable.
